# Gunn rats with glial activation in the hippocampus show prolonged immobility time in the forced swimming test and tail suspension test

**DOI:** 10.1002/brb3.1028

**Published:** 2018-06-28

**Authors:** Ryosuke Arauchi, Sadayuki Hashioka, Keiko Tsuchie, Tsuyoshi Miyaoka, Toshiko Tsumori, Erlyn Limoa, Ilhamuddin A. Azis, Arata Oh‐Nishi, Shoko Miura, Koji Otsuki, Misako Kanayama, Muneto Izuhara, Michiharu Nagahama, Kiminori Kawano, Tomoko Araki, Kristian Liaury, Rostia A. Abdullah, Rei Wake, Maiko Hayashida, Ken Inoue, Jun Horiguchi

**Affiliations:** ^1^ Department of Psychiatry Shimane University Izumo Japan; ^2^ Department of Nursing Prefectural University of Hiroshima Mihara Japan; ^3^ Department of Psychiatry Hasanuddin University Makassar South Sulawesi Indonesia; ^4^ Health Service Center Kochi University Kochi Japan

**Keywords:** astrocytes, forced swimming test, Gunn rat, hippocampus, microglia, tail suspension test

## Abstract

**Introduction:**

Recent studies imply that glial activation plays a role in the pathogenesis of psychiatric disorders, such as schizophrenia and major depression. We previously demonstrated that Gunn rats with hyperbilirubinemia show congenital gliosis and schizophrenia‐like behavior.

**Methods:**

As it has been suggested that major depression involves glial activation associated with neuroinflammation, we examined whether Gunn rats show depression‐like behavior using the forced swimming test (FST) and the tail suspension test (TST). In addition, we quantitatively evaluated both microgliosis and astrogliosis in the hippocampus of Gunn rats using immunohistochemistry analysis of the microglial marker ionized calcium‐binding adaptor molecule (Iba) 1 and the astrocytic marker S100B.

**Results:**

Both the FST and TST showed that immobility time of Gunn rats was significantly longer than that of normal control Wistar rats, indicating that Gunn rats are somewhat helpless, a sign of depression‐like behavior. In the quantification of immunohistochemical analysis, Iba1immunoreactivity in the dentate gyrus (DG), cornu ammonis (CA) 1, and CA3 and the number of Iba1‐positive cells in the CA1 and CA3 were significantly increased in Gunn rats compared to Wistar rats. S100B immunoreactivity in the DG, CA1, and CA3 and the number of S100B‐positive cells in the DG and CA3 were significantly increased in Gunn rats compared to Wistar rats.

**Conclusion:**

Our findings suggest that both microglia and astrocyte are activated in Gunn rats and their learned helplessness could be related to glial activation.

## 1 INTRODUCTION

Recent studies imply that glial activation plays a role in the pathogenesis of psychiatric disorders, such as schizophrenia (Bernstein, Steiner, Guest, Dobrowolny, & Bogerts, 2015; Catts, Wong, Fillman, Fung, & Shannon Weickert, 2014) and major depression (Stockmeier et al., 2004; Torres‐Platas, Cruceanu, Chen, Turecki, & Mechawar, 2014), even though very little is known about their pathophysiology. In particular, several postmortem brain studies indicate that activated microglia are involved in both schizophrenia (Steiner et al., 2006) and major depression (Bayer, Busleia, Havasb, & Falkaia, 1999; Steiner et al., 2011). In accordance with, microglial activation may be a common finding in the pathogenesis of both the psychiatric diseases.

So far, a number of studies have attempted to establish appropriate animal models for these psychiatric disorders. However, the numbers of established models which show signs or symptoms relevant to the diseases are limited. We previously demonstrated that Gunn rats with hyperbilirubinemia showed the activation of both microglia stained with CD11b (Furuya et al., 2013; Liaury et al., 2012, 2014; Limoa et al., 2016) and astrocytes stained with glial fibrillar acidic protein (GFAP) (Limoa et al., 2016) in the hippocampus and exhibited schizophrenia‐like behavior in the prepulse inhibition test (Liaury et al., 2014; Limoa et al., 2016), the object‐location test (Furuya et al., 2013), and the novel object recognition test (Liaury et al., 2014).

Increasing numbers of studies support the idea that major depression is a multifactorial disease with both genetic and environmental factors contributing to disease development (Shelton, 2007). Inflammatory processes may also play a role in the etiology of major depression, at least in a subset of susceptible individuals. In addition, circumstantial evidence suggests that major depression involves glial activation associated with neuroinflammation (Hashioka, Miyaoka, Wake, Furuya, & Horiguchi, 2013; Popa‐Wagner, Buga, Tica, & Albu, 2014). In fact, inflammatory cytokines such as interleukin (IL)‐2 used for cancer treatment and interferon‐α used for hepatitis C treatment are well known to induce depressive symptoms as side effects. Also in animal experiments, depressive behavioral abnormalities are observed in rats treated with lipopolysaccharide or pro‐inflammatory cytokines (Kelley et al., 2013; Yirmiya, 1996). Neuroinflammation can be considered to be a glial cell‐propagated inflammation (Hashioka, 2011). These observations prompted us to determine whether glial activated Gunn rats also show depression‐like behavior. We tested the presence of depression‐related behavior in Gunn rats using the forced swimming test (FST) and the tail suspension test (TST). In order to establish glial activation in Gunn rats, we quantitatively evaluated both microgliosis and astrogliosis in the hippocampus of Gunn rats using immunohistochemical analysis for the microglial marker ionized calcium binding adaptor molecule (Iba)1 and the astrocytic marker S100Β.

## 2 MATERIALS AND METHODS

### 2.1 Animals

Seven‐week‐old male homozygous (j/j) Gunn rats and male Wistar rats (Japan SLC, Kurume, Japan) were used in this study. The rats were housed under standard conditions with a room temperature (RT) of 23 ± 2°C, humidity of 55 ± 5%, and 12 hr light/12 hr dark cycle (light phase 7:00 to 19:00). All rats were given free access to food and water. Two weeks before starting the experiment, the rats underwent a handling procedure once daily to reduce stress during the experiments. Gunn rats and Wistar rats were separated into three groups (i.e., the forced swimming test group, the tail suspension test group, and the immunohistochemistry group). Each individual was given a single test. All experiments were performed with the approval of the Shimane University Animal Ethics Committee, under the guidelines of the National Health and Medical Research Council of Japan.

### 2.2 Forced swimming test

A previous protocol reported for the Forced swimming test (FST; Detke & Lucki, 1996) was modified and followed. In brief, Gunn rats (*N *= 6) and Wistar rats (*N *= 6) were placed into a plastic cylinder (diameter 19 cm) that was filled with 10L of water (depth 40 cm, 25 ± 1°C) for two successive days. On the first day, rats were placed in the water for 15 min for habituation, dried in a heater, and returned to their home cage. On the second day, rats were placed in the water for 6 min and their behavior was recorded with a video camera. Immobility time in the final 5 min was measured from the recorded video. The rats were considered immobile when they remained motionless and floated, and when they moved only to keep their heads above the water. The cylinder was washed, rinsed, and refilled with fresh water at 25 ± 1°C for every test.

### 2.3 Tail suspension test

A previous protocol reported for the TST (Yan et al., 2014) was modified and followed. In brief, cleaned tails of rats were wrapped in adhesive tape at approximately half distance from the base. An experimental clip (Yamashitagiken, Tokushima, Japan) was attached to the adhesive tape. Gunn rats (*N *= 6) and Wistar rats (*N *= 6) were suspended 30–40 cm above the floor by the clip and were videotaped for 5 min. Immobility time in the 5 min was measured from the recorded video. Immobility was defined as a lack of attempt to move their limbs and staying in the vertical posture during suspension.

### 2.4 Brain section preparation

Animals underwent deep intraperitoneal anesthesia with an anesthetic mixture of three drugs: medetomidine (Domitor, Nippon Zenyaku Kogyo, Tokyo, Japan), midazolam (Dormicum, AstellasPharma, Tokyo, Japan), and butorphanol (Vetorphale, Meiji Seika Pharma, Tokyo, Japan). We mixed medetomidine 0.15 mg, midazolam 2 mg, and butorphanol 2.5 mg/kg b.w./rat and added saline (Otsuka Pharmaceutical Factory, Tokushima, Japan) to adjust the mixture to a volume of 0.5 ml/100 g b.w./rat. The rats were perfused transcardially with saline, followed by 4% paraformaldehyde (PFA) in 0.1 M phosphate buffer (PB). The brains were taken out and were fixed with 4% PFA in 0.1 M PB at room temperature (RT) for 4 hr. The brains were immersed in 10% sucrose at 4°C overnight and subsequently were immersed in 20% sucrose at 4°C for 3 days. The brains were cut at 40 μm thickness with a freezing microtome (Microm HM 430; Thermo Scientific, Germany).

### 2.5 Immunohistochemistry for glial markers

We modified and followed the immunohistochemical procedure described in our previous study (Limoa et al., 2016). The free‐floating brain sections were incubated in 1% H_2_O_2_ for 30 min at RT and then were preincubated with 0.1 M PB containing 3% bovine serum albumin, 0.2% Triton X and 1.5% goat serum for 1 hr at RT. The sections were incubated overnight with the rabbit anti‐Iba1 antibody (1:4000, Wako, Osaka, Japan) at RT. Similar procedures were carried out using the rabbit anti‐S100B antibody (1: 1500). Afterwards, the sections were incubated for 1 hr with biotinylated anti‐rabbit IgG antibody (1:200, standard ABC kit, Vector Laboratories, CA, USA) at RT. The immunoreactivity in the sections was developed by incubating in phosphate‐buffered saline (PBS) containing 0.5% diaminobenzidine (DAB) and 0.03% H_2_O_2_ for 10 min. The DAB reaction was halted by PBS. The tissues were mounted onto gelatin‐coated slides and subsequently sunk in graded alcohol baths for dehydration. Coverslips (Matsunami Glass, Osaka, Japan) were applied onto the slides with mounting medium.

### 2.6 Image analysis

The immunoreactivity for Iba1 or S100Β in the DAB staining was examined under a BZ‐X700 All‐in‐One Microscope (Keyence, Osaka, Japan) with a 20x objective lens. Images were captured from three areas within the hippocampus, namely DG, CA1, and CA3. Twenty twenty‐seven images were taken bilaterally under a BZ‐X700 from each area (10–13 images from the left hemisphere and 10–14 images from the right hemisphere). Overall, 60–84 images per animal (*N *= 6) were analyzed. The percent area occupied by Iba1‐ or S100B‐immunopositive cells per view was measured and the number of Iba1‐ or S100B‐immunopositive cells within a view field was counted using the software BZ‐X analyzer (Keyence).

### 2.7 Measurement of tumor necrosis factor (TNF)‐ α

The amount of TNF‐α in the hippocampus was measured using an ELISA kit (Life technologies, KRC3011, Tokyo, Japan). Hippocampal tissues were isolated and homogenized in ice‐cold cell lysis buffer (Cell Signaling, #9803, Danvers, NA, USA) supplemented with protease inhibitor (Roche, 11873580001, Mannheim, Germany). The tissues were extracted at ratio of 100 mg of tissue to 1 ml of buffer. The tissues were sonicated and subsequently centrifuged at 13,000 *g* at 4°C for 10 min. The supernatants were collected and studied. ELISA was performed according to the manufacturer's instruction.

### 2.8 Statistical analysis

All the data are presented as the mean ± standard error of the mean (SEM). Differences between the Wistar rat group and the Gunn rat group were evaluated by independent sample t test. This analysis was performed with the SPSS software (Dr. SPSS II for Windows, IBM Japan, Tokyo, Japan). A *p*‐value <0.05 was considered statistically significant.

## 3 RESULTS

### 3.1 Behavioral tests

**Figure 1:**
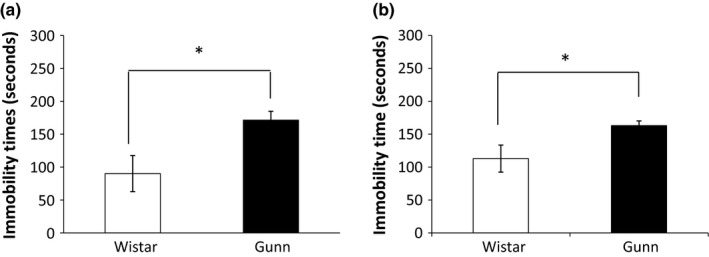
Immobility time of Wistar and Gunn rats in the FST and TST. Immobility time of Gunn rats is significantly longer than that of Wistar rats in both the FST (a) and the TST (b). Each value is the mean ± SEM (*N *= 6). *p < 0.05. FST, forced swimming test; TST, tail suspension test

We first evaluated the ability of rats to cope with stressful and inescapable situations, using both the FST and the TST. In these tests, immobility time indicates learned helplessness, which is a sign of depression‐like behavior (Cryan & Mombereau, 2004). In the FST, the mean immobility time of Wistar rats (*N *= 6) was 90.21 ± 27.40 s and that of Gunn rats (*N *= 6) was 171.23 ± 13.46 s (Figure 1a). The immobility time of Gunn rats in the FST was significantly longer than that of Wistar rats. No rat required rescue in the FST.

To further validate the helplessness of Gunn rats, the TST was also performed. In the TST, the mean immobility time of Wistar rats (*N *= 6) was 113.00 ± 20.57 s and that of Gunn rats (*N *= 6) was 163.57 ± 7.10 s (Figure 1b). Similar to the findings in the FST, the immobility time of Gunn rats in the TST was significantly longer than that of Wistar rats.

### 3.2 Immunohistochemistry

**Figure 2:**
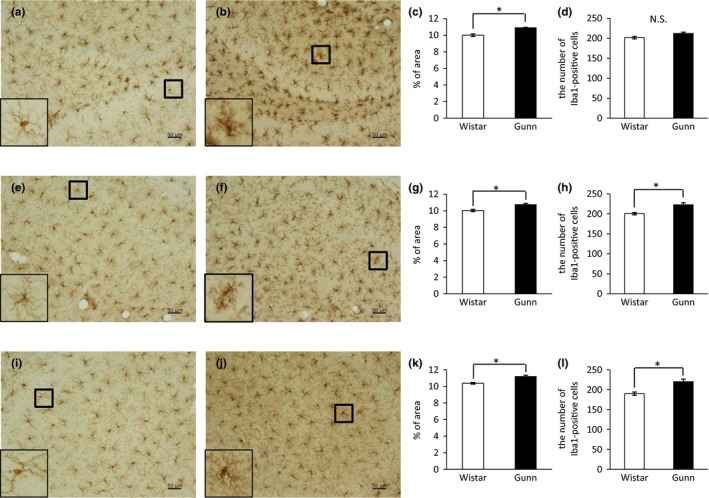
Immunoreactivity for Iba1 in the hippocampus. Representative images of DAB staining in the DG of Wistar (a) and Gunn rats (b), CA1 of Wistar (e) and Gunn rats (f), and CA3 of Wistar (i) and Gunn rats (j). The magnified pictures of Iba1‐positive cells are shown in the lower‐left insets. The scale bars indicate 50 μm. Quantification data for Iba1 immunoreactivity in the DG (c), CA1 (g), and CA3 (k). The number of Iba1‐positive cells in the DG (d), CA1 (h), and CA3 (l). Each value is the mean ± SEM (*N *= 6). *p < 0.05. N.S., not significant

We next evaluated the immunoreactivity for the microglial marker Iba1 in the DG, CA1, and CA3 regions of the hippocampus. In the DG, immunohistochemical images showed a higher expression of Iba1 in Gunn rats (Figure 2b) compared to Wistar rats (Figure 2a). A magnified image of an Iba1‐positive cell in Gunn rats showed an enlarged cell body with thick, shrunk processes (the lower‐left inset of Figure 2b), a picture which is consistent with the ameboid morphology of activated microglia. On the other hand, an Iba1‐positive cell in Wistar rats showed a thin cell body with fine and long processes (the lower‐left inset of Figure 2a), consistent with the ramified morphology of resting microglia. Quantification of data for Iba1 expression showed that the Iba1‐positive area was significantly increased in Gunn rats compared to Wistar rats in the DG (Figure 2c). Also in both the CA1 and CA3, we found that the Iba1‐positive area was significantly enlarged in Gunn rats compared to Wistar rats (Figure 2g, k). Many of the Iba1‐positive cells in Gunn rats displayed activated/ameboid morphology (the insets of Figure 2f, j), while many of those in Wistar rats exhibited the ramified morphology (the lower‐left insets of Figure 2e, i). We counted the number of Iba1‐labelled cells in the hippocampus. In the DG, there was no significant difference in the number of Iba1‐positive cells between Gunn rats and Wistar rats (Figure 2d). In the CA1 (Figure 2h) and CA3 (Figure 2l), we found, however, that the number of Iba1‐positive cells was significantly increased in Gunn rats compared to Wistar rats. As shown in magnified images, the area of each Iba1‐positive cell in the DG of Gunn rats (the lower‐left inset of Figure 2b) looks bigger than that of Wistar rats (lower‐left inset of Figure 2a). Therefore, the discrepancy between the Iba1 immunoreactivity and the number of Iba1‐positive cells in the DG may stem from the difference in the area of each Iba1‐positive cell.

**Figure 3:**
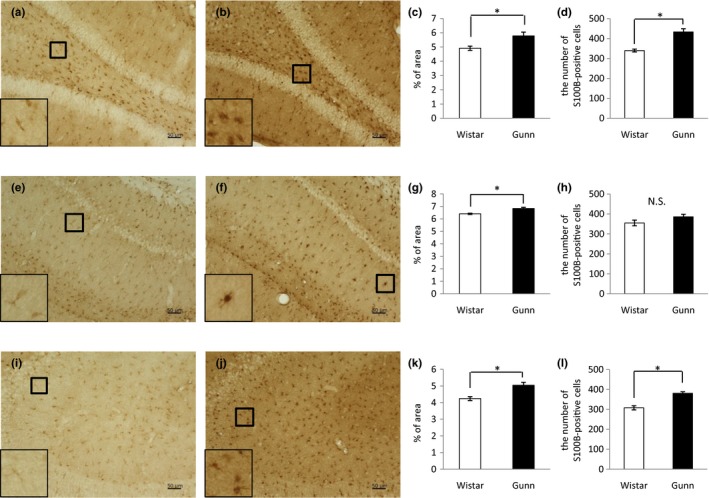
Immunoreactivity for S100B in the hippocampus. Representative images of DAB staining in the DG of Wistar (a) and Gunn rats (b), CA1 of Wistar (e) and Gunn rats (f), and CA3 of Wistar (i) and Gunn rats (j). The magnified pictures of S100B‐positive cells are shown in the lower‐left insets. The scale bars indicate 50 μm. Quantification data for S100B immunoreactivity in the DG (c), CA1 (g), and CA3 (k). The number of S100B‐positive cells in the DG (d), CA1 (h), and CA3 (l). Each value is the mean ± SEM (*N *= 6). *p < 0.05. N.S., not significant

We also examined the immunoreactivity for the astrocytic marker S100B in the hippocampal DG, CA1, and CA3. Representative images of Immunohistochemical analysis showed a higher expression of S100B in the DG (Figure 3b), CA1 (Figure 3f), and CA3 (Figure 3j) of Gunn rats compared to Wistar rats (Figure 3a, e, i). Quantitation of S100B immunoreactivity revealed that the S100B‐positive area in Gunn rats was significantly increased compared to Wistar rats in the DG (Figure 3c), CA1 (Figure 3g) as well as in the CA3 (Figure 3k). Magnified images showed that many of astrocytic cell bodies in Gunn rats were hypertrophic and stained intensely for S100B (the inset of Figure 3b, f, j), while many of those in Wistar rats looked like threads and stained weakly for S100B (the inset of Figure 3a, e, i). The numbers of S100B–positive cells in the DG, CA1, and CA3 were counted. Both in the DG (Figure 3d) and the CA3 (Figure 3l), the numbers of S100B–positive cells in Gunn rats were significantly greater than in Wistar rats. In the CA1, we observed the nonsignificant trend indicating that the number of S100B–positive cells in Gunn rats was more than in Wistar rats (Figure 3h).

### 3.3 Pro‐inflammatory cytokine quantification

**Figure 4:**
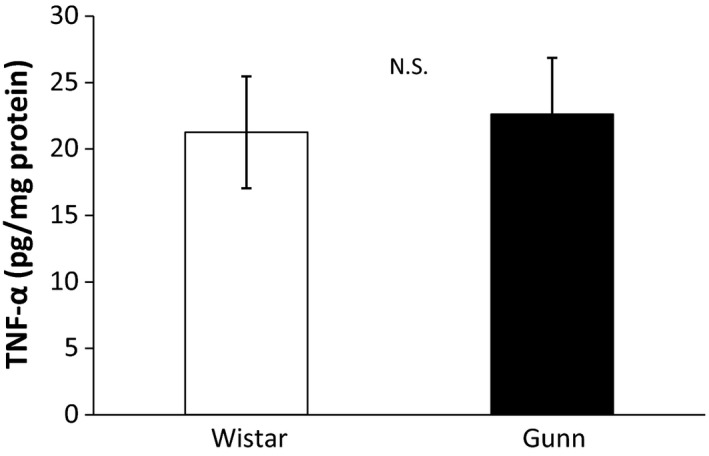
Quantification of TNF‐α in the hippocampus. The amount of TNF‐α in the hippocampus was measured using ELISA. There was no significant difference between Gunn rats and Wistar rats. Each value is the mean ± SEM (*N *= 8). N.S., not significant

We finally measured the amount of TNF‐α, a typical pro‐inflammatory cytokine, in the hippocampus to establish the presence of neuroinflammation in Gunn rats. The mean amount of TNF‐α in Gunn rats (*N *= 8) was 22.62 ± 4.23 pg/mg protein and that of Wistar rats (*N *= 8) was 21.26 ± 4.21 pg/mg protein (Figure 4). There was no significant difference between Gunn rats and Wistar rats.

## 4 DISCUSSION

The present study had three major findings. First, the immobility time of Gunn rats was significantly longer than that of normal control Wistar rats in both the FST and TST, suggesting that Gunn rats exhibit learned helplessness, a sign of depression‐like behavior. Second, the hippocampal immunoreactivity for Iba1 and the number of Iba1‐immunopositive cells in the CA1 and CA3 regions were significantly increased in Gunn rats compared to Wistar rats, indicating that hippocampal microglia of Gunn rats are activated. Third, the hippocampal immunoreactivity for S100B and the number of S100B‐immunopositive cells in the DG and CA3 regions were also significantly increased in Gunn rats compared to Wistar rats, demonstrating that hippocampal astrocytes of Gunn rats are activated.

The results of this study indicating that both microglia and astrocytes are significantly activated in the hippocampus of Gunn rats as shown by immunohistochemistry using Iba1 and S100B are consistent with our previous studies which demonstrated increased immunoreactivity for another microglial marker CD11b (Furuya et al., 2013; Liaury et al., 2012, 2014) and for another astrocytic marker GFAP (Limoa et al., 2016) in the hippocampal DG, CA1, and CA3. These observations of the increased immunoreactivity using two distinct markers for each glial cell substantiate both microglial activation and astrocytic activation in the hippocampus of Gunn rats. In this study, we intensively analyzed the hippocampus as the region of interest, based on the evidence that hippocampal dysregulation along with its small volume is strictly associated with major depression and stress (Kino, 2015; MacQueen & Frodl, 2011). Activated microglia and astrocytes may contribute to neuronal cell death in the hippocampus and result in hippocampal atrophy, as the activation of both types of glial cells has been demonstrated to cause neurotoxicity in vitro (Hashioka, Klegeris, & McGeer, 2011, 2012; Hashioka, Klegeris, Schwab, & McGeer, 2009).

The Gunn rat is a mutant of the Wistar strain and has a genetic deficiency in glucuronyltransferase (Chowdhury, Kondapalli, & Chowdhury, 1993; Gunn, 1944). This deficiency leads to high levels of unconjugated bilirubin in their blood and various tissues, including the brain. Therefore, Gunn rats have been used as an experimental model of kernicterus (bilirubin encephalopathy), which can be considered as an organic brain disorder. As patients with organic brain disorder often present symptoms of schizophrenia and affective disorder (Hamilton, Frick, Takahashi, & Hopping, 1983), it is not unexpected that Gunn rats show depression‐like behavior as demonstrated by this study and schizophrenia‐like behavior as shown by our previous studies (Furuya et al., 2013; Liaury et al., 2012, 2014; Limoa et al., 2016). It has been shown that a neuronal damage in the hippocampus is caused by unconjugated bilirubin in growing Gunn rats (Ohno, 1981). In addition, Gunn rats have been demonstrated to show impaired recognition memory (Liaury et al., 2014). Such damage of hippocampal neurons and impaired recognition memory in Gunn rats may parallel the clinical observation that depressed patients often manifest poor concentration and memory. It has been demonstrated that brain ischemia causes astrocytic activation and neuronal death in the CA1 and induces impairment of both reference and working spatial memory in rats (Cechetti et al., 2012). In accordance with, glial activation in the CA1, whose damage is sufficient to produce amnesia as shown in a case report of the patient RB (Zola‐Morgan, Squire, & Amaral, 1986), might cause a decline in memory through the hippocampal neuronal death due to the neurotoxicity of activated glial cells.

Although the immobility time in the FST represents desperate behavior associated with major depression, it can also be considered as a form of apathy or avolition, which is a part of the negative symptoms of schizophrenia. A comprehensive behavioral test battery is required in further studies to clarify whether Gunn rats have symptoms of the other psychiatric disorders. Unconjugated bilirubin has been shown to activate both microglia (Gordo et al., 2006) and astrocytes (Fernandes et al., 2011) in vitro. In accordance with, microglial activation and astrocytic activation in Gunn rats appear to be caused by high levels of unconjugated bilirubin, which can enter the brain as the free fraction (Ostrow, Pascolo, Shapiro, & Tiribelli, 2003).

Chronic mild stress has been shown to cause depression‐like behavior and induce the production of inflammatory cytokines such as IL‐1β and IL‐6 in rats. Antidepressant treatment attenuates the expression of inflammatory cytokines and depression‐like behavior of the stressed rats (Jiang et al., 2013; Rossetti et al., 2016). Based on these findings, inflammation may play a central role in the induction of a depressive phenotype. In order to establish the presence of neuroinflammation in Gunn rats, we measured the amount of TNF‐α in the hippocampus. Nevertheless, there was no significant difference in the hippocampal amount of TNF‐α between Gun rats and Wistar rats. It is unknown why Gunn rats did not show an increase in TNF‐α levels in the hippocampus. It is obviously needed in the future to determine whether the other neuroinflammatory molecules are up‐regulated in the hippocampus of Gunn rat.

It is unclear whether microglial activation is closely associated with the pathogenesis of major depression, as only a few studies have examined microglial activation in postmortem samples from patients who suffered from major depression. Steiner et al. (2008) demonstrated that the density of human leukocyte antigen‐DR‐immunoreactive microglia in the prefrontal cortex, anterior cingulate cortex, and mediodorsal thalamus was elevated in patients with major depression who committed suicide, while the microglial density was not increased in depressed patients who did not commit suicide. Steiner et al. (2011) also showed an increase in the density of quinolinic acid‐immunoreactive microglia in the anterior cingulate cortex of severely depressed individuals who committed suicide compared with matched controls. Torres‐Platas et al. (2014) showed that the ratio of activated microglia over ramified microglia and Iba1gene expression were significantly increased in depressed suicides compared to healthy controls. Although our observation that Gunn rats with microglial activation in the hippocampus show depressive behavior is basically in line with these postmortem studies on major depression, further investigations on this issue are clearly warranted.

The correlation between astrocytic activation related to the number/density of astrocytes and the pathogenesis of major depression is controversial. Our finding that Gunn rats with astrocytic activation in the hippocampus exhibit depressive behavior is consistent with a postmortem study by Stockmeier et al. (2004) who reported an increase in the density of glial cells in the hippocampus of major depression patients. Our result is also compatible with another postmortem study, which showed that older subjects with late‐onset depression had an increased astrocytic population in the dorsolateral prefrontal cortex (Miguel‐Hidalgo et al., 2000). On the other hand, several postmortem studies on major depression have given opposite findings, namely, prominent decrease in the number and the density of astrocytes in major depression patients compared to age‐matched nonpsychiatric controls. Such a reduction in astrocytic population was observed in the dorsolateral prefrontal (Cotter, Mackay, Landau, Kerwin, & Everall, 2001; Rajkowska et al., 1999), orbitofrontal (Rajkowska et al., 1999), subgenual (Ongur, Drevets, & Price, 1998), and anterior cingulate cortex (Cotter et al., 2001). This discrepancy may stem from a difference in brain regions studied.

Our observation that Gunn rats with both microglial activation and astrocytic activation show depression‐like behavior is not surprising, considering the fact that systematic administration of lipopolysaccharide in rodent leads to the activation of microglia and astrocytes, which is accompanied by sickness behavior, such as decreased activity, lethargy, loss of appetite, and reduced social interaction (Biesmans et al., 2013; Norden, Trojanowski, Villanueva, Navarro, & Godbout, 2016; Townsend, Chen, Jeffery, & Johnson, 2014). In addition, the olfactory bulbectomized rat shows both microglial activation and astrocytic activation as demonstrated by an increase in the mRNA expression of CD11b and GFAP (Burke et al., 2013). A manifestation of major depression symptoms may therefore be mediated, at least in part, by abnormal activation of both microglia and astrocytes. If this is correct, impaired behavior in Gunn rats will be ameliorated after inhibition of glial activation. Indeed, we have previously demonstrated that minocycline attenuates microglial activation in the hippocampus and improves impaired recognition memory in Gunn rats (Liaury et al., 2014). As it is becoming widely accepted that activated microglia act as immunoregulators of astrocytic activation, and *vice versa* (Hashioka et al., 2013; Liu, Tang, & Feng, 2011), studies of activation of microglia as well as astrocytes in the pathological brain may be very important in shedding light on the pathogenesis of psychiatric disorders.

## CONFLICT OF INTEREST

None declared.
